# Forecasting and analysing global average temperature trends based on LSTM and ARIMA models

**DOI:** 10.1371/journal.pone.0330645

**Published:** 2025-09-03

**Authors:** Can Tan, Junyi Zhong, Dajun Yang, Weiming Huang

**Affiliations:** 1 School of Management, Guangdong Ocean University, Zhanjiang City, Guangdong Province, China; 2 School of Business, Shandong University, Weihai City, Shandong Province, China; 3 School of Mathematics and Computer Science, Guangdong Ocean University, Zhanjiang City, Guangdong Province, China; 4 School of Administration, North Sichuan Medical College, Nanchong City, Sichuan Province, China; 5 Faculty of Foreign Languages, Guangdong Ocean University, Zhanjiang City, Guangdong Province, China; Khalifa University, UNITED ARAB EMIRATES

## Abstract

Previous studies have demonstrated a significant correlation between global average temperature change trends and greenhouse gases, and employed various prediction models. However, the potential of the combination of the LSTM and ARIMA models for temperature forecasting has not been fully explored, especially in terms of enhancing prediction accuracy. Based on the hypothesis that COVID-19 has affected the global average temperature, this study utilizes global average temperature data from 1880 to 2022. We combine the LSTM model, which excels at capturing long-term dependencies, with the ARIMA model, known for its effectiveness in handling linear time series data, to predict the global mean temperature. This combination compensated for the limitations of individual models, providing a more accurate and comprehensive temperature forecast. Our findings reveal that the early trend of global temperature rise is significant, yet the implementation delay leads to severe issues. Moreover, COVID-19 has indirectly reduced greenhouse gas emissions, slowing global warming. Additionally, we find that the correlation between longitude and mean temperature is weak, while the correlation between latitude and temperature is strongly negative. This study offers valuable insights and provides a reliable prediction method for ecological environment governance and the formulation of economic construction policies.

## 1. Introduction

The global average temperature is a crucial indicator of global warming [1]. In addition to indicating climate trends and influencing agricultural production, the global average temperature plays a vital role in water resource management, sea level change, and predicting extreme weather events [2]. As a result, many researchers have historically invested their efforts in studying the mechanisms and impacts of global temperature change to address the environmental and social issues associated with climate change [3,4]. However, in recent years, industrialisation, urbanisation, and the low penetration of new energy technologies have posed significant challenges to mitigating global warming [5]. Therefore, studying the factors that influence global temperature change and their trend prediction is highly significant. However, until now, scholars in environmental science research have left a gap in studying the interaction between global temperature change and multi-gas synthesis, multi-scale time series, and regional climate response.

Our reading of the literature reveals that the issue of global warming has achieved widespread attention [6]. For example, the importance of linking extreme regional temperature changes to global warming levels to understand the impact of global emission targets on regional climate is discussed in a study by Sun, Yu [7]. However, the above studies have not focused on the complex mechanisms of global temperature warming and the problem of accurately predicting future trends. In a recent article, however, researchers have focused on the use of CMIP6 models for assessing and predicting temperature datasets [8]. GHGs are considered well-mixed in IPCC reports, and most studies utilize CMIP models [9,10]. However, these studies did not examine the effects of single or mixed greenhouse gases on global temperatures.

Over the past 160 years, studying global temperature change has been a research hotspot for many scholars [11]. For example, Meehl, Teng [12] noted that in the second half of the 21st century, global warming trends are expected to become more significant, frequent, and prolonged. Specifically, scholars have explored the impacts of global temperature change from various perspectives, including climate change, ecosystem response, and economic development [4]. For example, Mann, Bradley [13] revealed the trend of global temperature change by analysing long-term temperature records. Although the above studies have focused on global temperature trends, few have examined the impact of global warming on socioeconomic activities, particularly the lack of studies on the effect of the COVID-19 pandemic on global temperature. The correlation between COVID-19 and global temperature has been largely confirmed in previous studies. For example, the study by Le Quéré, Jackson [14], the study provides a detailed analysis of the significant reduction in global CO_2_ emissions due to lockdowns and restrictions during the COVID-19 pandemic. This changing greenhouse gas can absorb and re-radiate the long-wave radiation emitted by the Earth’s surface, thereby raising the temperature of the Earth’s surface [15]. When emissions are reduced, the concentration of greenhouse gases in the atmosphere grows at a slower rate, and less additional heat enters the Earth’s system, slowing the rate at which global temperatures rise [16]. This study aims to fill this gap by providing insights into the trend of global temperature change and its complex relationship with socioeconomic activities through LSTM and ARIMA models combined with correlation analysis. In particular, we will analyse the potential impacts of reduced human activities during the COVID-19 pandemic on global temperature change.

Specifically, we use the LSTM and ARIMA models for the study and combine the Pearson and Spearman correlation coefficients for correlation analysis. The models and research methods employed in the paper have proven practical and scientifically sound in various fields, including weather forecasting and environmental science [17,18]. Based on this, we input the global average temperature data from 1880 to 2022 into the LSTM and ARIMA models to analyse and predict future global temperature changes. First, we preprocessed the global temperature data using normalization and five-fold cross-validation. Second, we constructed LSTM and ARIMA models for the prediction analysis of global temperature change. Finally, we explored the interrelationships between global temperature change, latitude, longitude, greenhouse gases, and COVID-19 through correlation analysis.

The present study contributes to studying global average temperature change and greenhouse gas emissions. Although the relationship between global temperature change and greenhouse gas emissions has been widely recognised, this study is an early attempt to incorporate the special socio-economic events of the COVID-19 pandemic, which provides a new perspective for understanding global temperature change. Second, this study can provide more accurate prediction models for research in the field of climate change, and it provides new research avenues for assessing the impacts of socioeconomic activities on global temperature change. In addition, this study finds a precise correlation between global temperature, latitude, and longitude, as well as greenhouse gases, which provides new data support for the study of regional climate change impacts. Ultimately, this study presents a novel theoretical framework for the multifaceted analysis of global temperature change, integrating various advanced statistical and machine learning methods. At the level of practical management, the results of this study can serve as a reference for policymakers to make more informed and rational decisions when formulating responses to climate change, particularly in light of the complexity of the impacts of economic and social activities on the climate.

## 2. Literature review

### 2.1. Global warming

Global warming refers to a sustained increase in the Earth’s surface temperature resulting from increased greenhouse gas emissions [19]. Global warming persists with events that affect the global climate system, change precipitation patterns, and increase the frequency of extreme weather events [20]. These events primarily occur due to the emissions of greenhouse gases, whose increased concentrations originate mainly from the burning of fossil fuels, agricultural activities, and land-use changes [21]. Since the Industrial Revolution, human activities have significantly increased the emissions of these gases, resulting in a substantial rise in atmospheric concentrations of greenhouse gases [22].

Global temperatures have risen significantly over the past century, and this trend has become more pronounced, especially in the past few decades [23]. This has had wide-ranging impacts on, among other things, ecosystems, agriculture, and public health. For example, there has been an increase in the frequency and intensity of extreme weather events, such as heatwaves, heavy rainfall, and droughts, which pose enormous challenges to human societies and the natural environment [24]. Additionally, the melting of glaciers and ice caps, as well as the thermal expansion of the oceans, has led to rising sea levels, which threaten coastal ecosystems and human settlements [25]. Therefore, to mitigate the risks mentioned above, people around the globe are increasingly aware that reducing greenhouse gas emissions is crucial. This can be achieved by promoting clean energy and energy efficiency, as well as developing low-carbon technologies, such as adopting clean energy, which is estimated to reduce GHG emissions by about 30% in the Lima, Mendes [26] study. Secondly, the development and application of carbon capture and storage (CCS) technology, which captures and stores carbon dioxide emitted by industry underground, can reduce the concentration of carbon dioxide in the atmosphere [27]. Finally, we have an international perspective, where countries collaborate to reduce greenhouse gas emissions through international frameworks, such as the Paris Agreement [28].

The outbreak of COVID-19 in 2019 has had a significant impact on the global economy, health, environment, and other fields [29–31]. According to the World Meteorological Organization, the temperature on Earth has increased by nearly 1 °C since the beginning of industrialization. Global temperatures will rise 3–5 °C by 2100 [32]. Therefore, we believe that the problem of increasing global average temperature is becoming increasingly severe, and it is urgent to put forward measures to solve global warming.

### 2.2. Global average temperature

The global mean temperature is the average of the temperatures of all land and ocean areas on the Earth’s surface over a given period [33]. Understanding climate change, predicting the weather, and guiding human activities are essential [34]. As one of the critical indicators of the state of the Earth’s climate, global average temperature monitors climate change, assessing impacts on ecosystems, and validating climate models. Studies have found that the current global average temperature shows a significant upward trend [35]. This phenomenon is generally attributed to increased greenhouse gas emissions, global industrialisation, and natural climate variability [36].

In the field of environmental sciences, numerous studies have been conducted on the global average temperature, and it has gradually become a new focal point for climate change research [37]. For example, the effect of atmospheric carbon dioxide on the global average temperature was first proposed by Arrhenius [44]. Several studies in different fields have been carried out around the globe in future studies; for example, Hansen, Sato [39] analysed the trend of global warming in the 20th century by using global temperature data; in the study of Routson, Kaufman [40] analyzing the paleoclimate records since the Holocene, it was found that the increase in the global average temperature in the 20th century was the largest in the past 10,000 years. Although previous studies have discussed the relationship between global average temperature and global warming, the link between the two has not been further analysed with data. In recent years, studies on climate change attribution have become a research hotspot, and the relationship between the global average temperature and its variations has gradually emerged [41]. In Climate Change 2023, the synthesis report of the Sixth Assessment Report (AR6), released by the United Nations Intergovernmental Panel on Climate Change (IPCC), explores the relationship between greenhouse gas (GHG) emissions and the rise in global average temperature in depth. The results show a positive correlation between GHG emissions and the increase in global average temperature. Many scholars have focused on the relationship between the global average temperature and global warming, and have further discussed how the global average temperature plays a significant role in indicating, driving, and influencing global warming [42]. Precisely, the global average temperature not only directly reflects the trend and extent of global warming but also serves as a central variable in predicting future climate change, assessing the impacts of human activities on the climate system, and influencing biodiversity. For example, the study of Kaufman, McKay [43] reflected and predicted the global warming trend using five different statistical methods for global average temperature. Therefore, this study holds significant practical value and theoretical importance, as it focuses on global average temperatures, future climate change projections, and the complexity of socio-economic activities.

In environmental science, research on the global average temperature began very early. For example, in the study of Valone [38], the global average temperature was first mentioned as a factor in assessing the degree of global warming. In future research, studies will be conducted across various fields of study to examine the global average temperature. For example, Chang, Mi [45] found that climate change may affect the GDP growth rate by changing the time resolution of temperature variables, introducing nonlinearity and specifying temperature bias into the econometric function; In the study of Marcott, Shakun [46], through the analysis of the paleo climatological record since the Holocene, it was found that the global average temperature increase in the 20th century was the largest in the past 10,000 years. In recent years, research on the attribution of climate change has become a research hotspot, and the relationship between global average temperature and climate change has gradually emerged. In Climate Change 2023, a comprehensive report of the sixth assessment report released by the United Nations IPCC [47,48], the relationship between GHG emissions and global average temperature rise is deeply explored, and the results show that there is a positive correlation between greenhouse gas emissions and global average temperature rise. Many scholars have focused on the relationship between the global average temperature and global warming, and have further discussed how the global average temperature plays a significant role in indicating, driving, and impacting global warming. Specifically, global average temperature can not only directly reflect the trend and degree of global warming but also be used as a core variable to predict future climate change and evaluate the impact of human activities on the climate system and biodiversity. For example, Kaufman, McKay [43] employed five different statistical methods to analyze and predict the global average temperature trend, reflecting the trend of global warming. This study reveals the severity of global temperature change and provides a scientific basis for developing strategies to mitigate climate change. Therefore, this study focuses on the global average temperature and the prediction of future climate change, as well as the examination of the complexity of social and economic activities, which has both crucial practical significance and theoretical value.

## 3. Materials and methods

### 3.1. Data sources

The data for this study were obtained from the global average temperature data from 1880 to 2022 on the NOAA website [49,50]. The global average temperature data from 1880 to 2022 was conditionally filtered through the website. Then, the data were exported to Excel to facilitate access to the data by the model and the machine learning model.

### 3.2. Experimental methods

The ARIMA model was first proposed and developed by Box and Jenkins in 1970 [51]. The ARIMA model is a commonly used time series analysis method for predicting future numerical data. The ARIMA model can be used to indicate the trend of the data in future periods by fitting the historical time series data. It is widely used in economics, finance, meteorology, medicine, and other fields. Du Preez and Witt [52] argued that the ARIMA model performs better than multivariate models. Goh and Law [53] confirmed that the ARIMA model is superior to the smoothing technique and other models for prediction estimation. Alsharif, Younes [54] also asserted that ARIMA is a suitable choice and a fluent technique when the data under study is distributed over a long period and the correlation of past observations is strong.

The LSTM model was born in 1997. It was proposed by Sepp Hochreiter and Jürgen Schmidhuber [55]. LSTM is a variant of Recurrent Neural Network (RNN), which solves the problems of gradient vanishing and gradient explosion in traditional RNNs by introducing a gating mechanism. The structure of LSTM enables it to capture long-term dependencies more effectively when dealing with time series data, thereby improving its ability to handle modeling and prediction tasks for long series data. Therefore, LSTM has numerous applications in finance, meteorology, ecology, and industrial production, among others. Xu, Pan [56] proposed a multi-input and stochastic-error-based LSTM model to predict dam settlement. The results demonstrate that the model outperforms general prediction models, such as support vector regression and multilayer perceptron models [57,58]. Thus, it is concluded that LSTM has higher accuracy and reliability than general prediction models.

Other machine learning methods, such as CNN and hybrid models (CNN combined with RNN), have certain drawbacks for this research domain. Although the CNN model (convolutional neural network model) has achieved great success in image recognition, natural language processing, and other fields [59], it has certain limitations when dealing with pure time series data. CNNS are mainly good at capturing local spatial features of data, and are relatively weak at processing long-term dependencies and time order information in time series [60]. In addition, CNNs usually require a large amount of labeled data for training, which may be limited in some time series prediction tasks, thereby limiting the performance of CNNs [61,62]. The hybrid model, which combines CNN and RNN as an example, can take into account both spatial features and time series modeling to a certain extent. However, its complexity is high, training is difficult, and it is prone to overfitting. Moreover, hybrid models require more expertise and experience to build and tune, and may not necessarily outperform LSTM or ARIMA models alone for domain-specific time series prediction tasks [63,64].

In this paper, we primarily utilize ARIMA and LSTM models to predict global temperature fluctuations and greenhouse gas emissions, as well as to examine the impact of the global COVID-19 outbreak on global temperature fluctuations and greenhouse gas emissions.

Firstly, this paper preprocesses the data, where the original temperature data are normalized to the interval [0, 1] using the Min-Max method, thereby eliminating dimensional influence between different units and improving the model’s convergence efficiency. Then, a five-fold cross-validation strategy is applied to prevent overfitting and enhance the model’s generalization ability. Secondly, the construction and application method of the LSTM model. One is to construct a standard LSTM structure with input gate, forget gate, output gate, and cell state update mechanism. The other is to take the processed time series data as input and use the least squares method as the loss function for training: $\text{L}\left(\text{y},\text{f}(x,\omega )\right)=\sum_{\text{i}=\text{1}}^{\text{m}}{\left[{\text{y}}_{\text{i}}-\text{f}({\text{x}}_{\text{i}},\omega_{\text{i}})\right]}^{\text{2}}$. Third, the gradient descent algorithm is used to iteratively optimize the model parameters until the test set loss function converges. The final model regressed and fitted the data from 1880 to 2022, and predicted the future temperature trend (12.79 °C in 2050 and 14.51 °C in 2100). Subsequently, the ARIMA model is modeled, and the implementation process is described. The first step is to perform an ADF stationarity test on the original sequence and determine that it is a non-stationary sequence before proceeding with first-order differencing. Secondly, the ACF/PACF plot is used to determine the model order (p, d, q), and the Ljung-Box white noise test is used to verify whether the residuals are white noise. The third is to construct the model in the form of: ${\text{y}\prime }_{\text{t}}=\alpha_{\text{0}}+\sum_{\text{i}=\text{1}}^{\text{p}}\alpha_{\text{i}}{\text{y}\prime }_{\text{t}-\text{1}}+\epsilon_{\text{t}}+\sum_{\text{i}=\text{1}}^{\text{q}}\beta_{\text{i}}\epsilon_{\text{t}-\text{1}}$. Finally, the future temperature is predicted and compared with the LSTM results (the expected temperature in 2050 is 12.05°C, and the predicted temperature in 2100 is 12.45°C). In this study, the fitting effect and prediction performance of the two models are compared, using indicators such as R^2^, RMSE, and MSE. Ultimately, LSTM is selected as the primary prediction model, as it is more suitable for modeling the complex, nonlinear trends in global temperature. At the same time, LSTM and ARIMA are widely used in environmental science and climate prediction, and have been demonstrated to have high accuracy and adaptability in numerous studies [65].

### 3.3. Data pre-processing

In the Long Short-Term Memory (LSTM) model, data Normalization is an essential data preprocessing step. Data normalization significantly improves the training efficiency and performance of the model by transforming the input data to a unified scale (such as mean 0 and variance 1), which not only accelerates the convergence speed of the model, but also helps to improve the generalization ability of the model, reduce the tendency of the model to overfit certain features, and enable the model to capture the long-term dependence in the data better. Therefore, this study primarily employs the Min-Max normalization method to preprocess the data for the LSTM model. Its formula ${\text{X}}_{\text{norm}}\backslash$ is shown in the following formula (1).


$$X_{norm}=\frac{X-X_{min}}{X_{max}-X_{min}}$$ (1)

In addition, this paper treats the latitude and longitude data by replacing the northern latitude with positive values, the southern latitude with negative values, the eastern longitude with positive values, and the western longitude with negative values and averaging the temperatures for each year for all regions of the country.

In addition, based on the experience of previous research, considering that global temperature data may be affected by seasonal changes and extreme climate events, and there are data integrity issues, this paper further reviews and processes the data quality. First, this paper uses the official NOAA annual global average temperature data from 1880 to 2022. The dataset is based on the integration of global land surface stations and ocean temperature grid data. It has been processed by monthly data weighting, spatial interpolation, and error control to eliminate noticeable seasonal fluctuations. Therefore, in this study, the data itself does not exhibit a periodic seasonal component and can be used directly for trend analysis without further seasonal adjustment or the construction of a seasonal modeling structure [66,67]. Secondly, due to the disturbance problem caused by extreme climate events such as volcanic eruptions and El Niño, this study focuses on the long-term trend modeling of temperature. It does not introduce a special elimination mechanism or dummy variable setting. In the modeling process, the marginal impact of such short-term anomalies on the temperature series can be automatically absorbed through the deep learning of long-term dependence features of the LSTM model, to retain the actual trend characteristics and improve the robustness of the prediction [68,69]. Finally, in terms of missing values, NOAA has verified that the data set used in this study has completed comprehensive quality control and missing value tests and processing before release, and there is no obvious problem of missing values or data fragmentation. Therefore, no additional interpolation or filling operations are needed, and the sequences used for model training have good continuity and integrity.

### 3.4. Splitting the training and test sets

When training neural networks, the data is often overfitted. The test set is data independent of training, not involved in training, and mainly used to evaluate the final model. Therefore, this paper proposes a five-fold cross-validation approach to address the above overfitting problem effectively.

This is primarily due to five-fold cross-validation, which aims to improve model generalization performance and evaluation accuracy. The training data is divided into five equal parts; one subset is used as the validation set, and the remaining four subsets are used as the training sets in turn. The five models are trained, and the average error is calculated to optimize the model parameters and evaluate the model’s robustness. Five-fold cross-validation effectively utilizes the limited data, and its evaluation results are as close as possible to the model’s performance on the test set, which can serve as an indicator for model optimization. The effectiveness of this method has been widely verified in environmental science and meteorological modeling [56,70,71].

### 3.5. Model construction

(1) LSTM model

This model is a type of feedback neural network; the core of its algorithm is to control the discarding or addition of information through the “gate,” thereby realizing the functions of forgetting and remembering. The internal structure comprises three gate structures: the input gate (it), the output gate (ot), the forgetting gate (ft), the cell state Ct, and the cell state update value Ct. The gate structure is equivalent to an information valve, which determines how much information can pass through it.


$$i_{t}=\sigma (W_{i}\left[h_{t-1},X_{t}\right]+b_{i})$$ (2)

$$o_{t}=\sigma (W_{o}\left[h_{t-1},X_{t}\right]+b_{o})$$ (3)

$$f_{t}=\sigma (W_{f}\left[h_{t-1},X_{t}\right]+b_{f})$$ (4)

where ${\text{W}}_{\text{i}}$ is the neuron weight of the i layer, ${\text{b}}_{\text{i}}$ is the bias, and σ(x) is the activation function. Where ${\text{W}}_{\text{i}}$ is the weight, ${\text{b}}_{\text{i}}$ is the bias, and since the model can optimize to get the best value by self-learning when performing network training, σ(x) is the activation function, and the output value is in the range between [0, 1], its mathematical expression is:


$$\sigma (x)=\frac{1}{\left(1+e^{-x}\right)}$$ (5)

The LSTM layer can jointly calculate the updated value of the cell state Ct at this time based on the network output at the previous moment and the network input at the current moment, which in turn combines the forgetting gate with the input gate to calculate the current cell state Ct, and thus its mathematical expression is:


$$\tilde{C_{t}}=\text{tan}\left(W_{c}\left[h_{t-1},X_{t}\right]+b_{c}\right)$$ (6)

$$C_{t}=f_{t}C_{t-1}+i_{t}\tilde{C_{t}}$$ (7)

$$\text{tan}\;h(x)=\frac{\left(e^{x}-e^{-x}\right)}{\left(e^{x}+e^{-x}\right)}$$ (8)

where $b_{c}$ is the bias of the cell state.

After setting up the LSTM model, the setup sample $\text{y}=\{{\text{y}}_{\text{1}},\;{\text{y}}_{\text{2}},.....{\text{y}}_{\text{m}}\}$ input into the LSTM neural network and apply the least squares method to calculate the loss function value of the training model


$$L\left(y,f(x,\omega )\right)=\sum {\mathrm{\backslash nolimits}}_{i=1}^{m}{\left[y_{i}-f(x_{i},\omega_{i})\right]}^{2}$$ (9)

The values that do not meet the requirements are corrected by the gradient descent algorithm, Omega, after the corrected xω\) is again fed into the LSRM neural network for operation. Then, the loss function in the test set training model meets the requirements, and the model is successfully trained. If the loss function in the test set training model does not meet the criteria, re-training is performed, and the training is completed.

(2) ARIMA model

The full name of this model is autoregressive moving average model, in which ARIMA (p, d, q) is called differential autoregressive moving average model, AR is autoregressive, p is autoregressive term; MA is moving average, q is the number of moving average terms, and d is the number of differences made when the time series becomes smooth. The core idea of the algorithm is to consider the data series of the predicted object over time as a random sequence and to approximate this sequence with a particular mathematical model. This model, once recognised, can be used to predict future values from past values of the time series, as well as the present values.

The ARIMA model is constructed in four steps: a smoothness test, a white noise test, a model test, and ARIMA model specification. We first collect comprehensive and relevant data, test the smoothness of the data through the Augmented Dickey-Fuller (ADF) test, determine the model order using the autocorrelation function (ACF) and partial autocorrelation function (PACF), and then estimate the model parameters. We iteratively adjusted the model parameters during the model fit testing phase to improve the data fit. Subsequently, we use the model to make predictions and compare them with actual observations for validity testing, ensuring the accuracy and reliability of the projections. This process demonstrates the applicability of the ARIMA model in time series forecasting and its flexibility and robustness in capturing dynamic changes in the data. The ARIMA model we chose is expressed as:


$${y\prime }_{t}=\alpha_{0}+\sum {\mathrm{\backslash nolimits}}_{i=1}^{p}\alpha_{i}{y\prime }_{t-1}+\varepsilon_{t}+\sum {\mathrm{\backslash nolimits}}_{i=1}^{q}\beta_{i}\varepsilon_{t-1}$$ (10)

**Fig 1:**
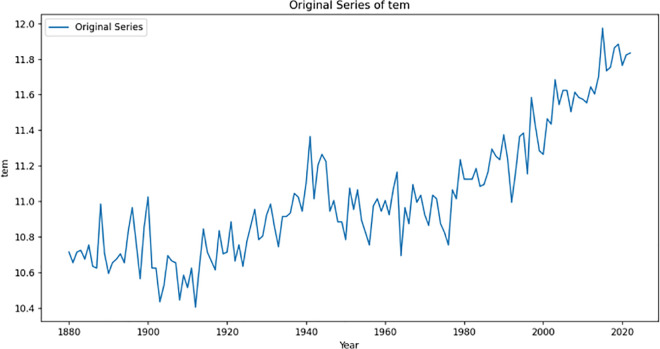
Original sequence diagram.

By obtaining and observing the original sequence plot (Fig 1), it is found that the data in the original sequence plot shows an upward trend, so it can be preliminarily judged that the original sequence plot is an unsteady sequence.

To test whether the original sequence diagram is a stationary sequence, we apply the ADF unit root test to evaluate the stationarity of this sequence diagram. The Augmented Dickey-Fuller Test (ADF) is a statistical test used primarily to determine whether time series data exhibit stationarity. Stationarity means that the statistical properties (such as mean, variance, autocovariance) of time series do not change with time, which is a crucial premise assumption of many time series analysis models (such as ARIMA, etc.).

The ADF test regression model is given by the following formula:


$$\Delta {\text{y}}_{\text{t}}=\alpha +\beta t+\gamma {\text{y}}_{\text{t}-\text{1}}+\sum {\mathrm{\backslash nolimits}}_{\text{i}=\text{1}}^{\text{p}}\delta_{\text{i}}\Delta {\text{y}}_{\text{t}-\text{i}}+\in_{\text{t}}$$ (11)

where ${\text{y}}_{\text{t}}$ is the observed value of the time series at time, $\Delta {\text{y}}_{\text{t}}$ is the first difference of ${\text{y}}_{\text{t}}$, and $\Delta {\text{y}}_{\text{t}}={\text{y}}_{\text{t}}-{\text{y}}_{\text{t}-\text{1}}$ and a is the constant term in the regression model (models two and three), which represents the basic level of the series. β is the coefficient of linear trend (model 3), reflecting the linear trend of the series; γ tests the key parameters, and the null hypothesis ${\text{H}}_{\text{0}}:\gamma =\text{0}$ indicates that the series has a unit root (non-stationary), and ${\text{H}}_{\text{1}}:\gamma <\text{0}$ indicates that there is no unit root (stationary). p lag order, the number of lagged difference terms in the regression model. If < p significance level (for example, 0.05), the null hypothesis is rejected and the series is considered stationary. If p ≥ significance level, the null hypothesis cannot be rejected, and the series is considered non-stationary. δ lags the coefficient of the difference term $\Delta {\text{y}}_{\text{t}-\text{i}}=(\text{i}=\text{1},\text{2},...,\text{p})$, which captures the series autocorrelation. $\in_{\text{t}}$ random error terms, independently and identically distributed, with mean 0 and constant variance.

We assume that the series is non-stationary and analyse it by the ADF test table, as shown in Table 1. The results show that without difference processing, the p-value is 0.169, indicating that the null hypothesis cannot be rejected. This suggests that the series is non-stationary and requires transformation into a stationary series through differencing. After first-order splitting, the p-value drops below 0.000, which means that the null hypothesis can be rejected, and the data after first-order splitting can be rejected at this time because the sequence has reached a plateau.

**Table 1:** Stability ADF test table.

ADF List of inspection						
Variable	Difference order	ADF Statistic	P	Critical value		
				1%	5%	10%
Tem	0	−2.3063	0.169	−3.478	−2.883	−2.578
	1	−11.6714	0.000***	−3.478	−2.883	−2.578
	2	−6.1874	0.000***	−3.483	−2.884	−2.579

Note: ***, **, and * represent 1%, 5%, and 10% significance levels, respectively.

At the same time, the autocorrelation function (ACF) and partial autocorrelation function (PACF) tests are used to further prove the stationary situation of the time series after the first difference. Where the Autocorrelation Function (ACF) measures the correlation between a time series and different lagged versions of itself, the Partial Autocorrelation Function (PACF) measures the direct correlation of a series with a lag after controlling for intermediate lags. ACF and PACF also determine the parameters p (autoregressive order) and q (moving average order) in ARIMA.

**Fig 2:**
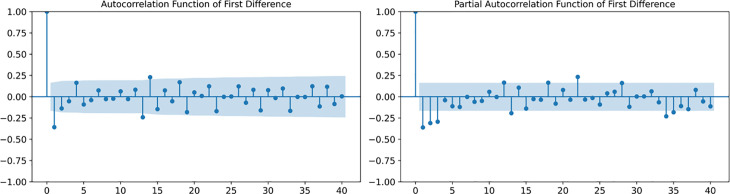
Autocorrelation Function (ACF) and partial autocorrelation function (PACF) plots.

By observing the autocorrelation coefficient and partial autocorrelation coefficient in the ACF and PACF graphs, the ACF value and PACF value are in shock and tend to 0 after the first order, which indicates that the time series after the first order difference is stationary. And by observing that the autocorrelation coefficient of ACF is close to 0 at the third order, then p = 3, and the autocorrelation coefficient of PACF is close to 0 at the fourth order, then p = 4. Combined with ADF test, after the first difference, p value drops to 0.000, which means ARIMA (3,1,4) is chosen as the training parameter of ARIMA model in this study. The following ACF and PACF plots are shown in Fig 2.

The white noise test was then performed on the data after first-order differencing by the Ljung-Box test. The test results indicate that the series is a non-white noise series (p < 0.05). Through the above steps, it is obtained to carry out the first-order differencing sequence is initially a non-smooth, non-white noise sequence, which is suitable for problem prediction using the ARIMA model, and the model test data are shown in Table 2. which yields an R^2^ close to 1, with a high degree of goodness of fit.

**Table 2:** Model test data table.

		Simulation degree of fit statistics		Yang Box Q [18]			
Model	Number of predictive variables	Stationary R-squared	R square	Statistical	DF	Significance	Number of outliers
Tem- Model_1	0	0.72	0.86	18.454	17	0.361	0

(3) Independent sample t-test

The independent sample t-test is used to determine whether there is a statistically significant difference between the means of two independent samples. It is suitable for analyzing the differences between the experimental group and the control group, as well as between different gender groups, in continuous variables (such as grades and reaction times).

In this study, the non-epidemic temperature samples from 2000 to 2019 were compared with the first-order difference samples of global temperature during the epidemic period from 2020 to 2022, and the independent sample t-test was performed. The table data showed that the result of Welch’s t-test was T = 0.77, P = 0.494. Although the P-value is greater than 0.05, a discernible trend is evident in the mean difference and effect size. Based on the existing sample data, the mean difference is 0.051, and Cohen’s d value is 0.392, suggesting a moderate effect size. It can be seen that although the impact of the epidemic on the increase in global temperature does not reach an obvious level, the mean difference and Cohen’s d value indicate that the epidemic has a certain impact on the rise in global temperature. The results are shown in Table 3.

**Table 3:** Independent sample t-test table after the first difference of global temperature.

Variable name	Variable value	M	SD	Mean difference	Cohen’s d value	Welch’s T
Temp_diff	During the pandemic	0.034	0.138	0.051	0.392	T = 0.77 P = 0.494
	Before the pandemic	−0.017	0.093

(4) Correlation analysis method

Correlation analysis is a method of plotting data samples into a scatterplot, and by observing the scatterplot, it is known whether there is a correlation between two samples so that it can be concluded whether the variables affect each other. This is done by testing the normality of the sample data and selecting the appropriate correlation coefficient. Testing the normality of the sample data can be done by examining the P-P plot or Q-Q plot to obtain the result. Through the normality test, the appropriate correlation coefficients are selected for analysis, where different coefficients correspond to various scenarios of use, and their effects are also different. For example, when applying the Pearson coefficient to Quantitative data, if the data meet the normality assumption, the normality plot indicates normality, and the scatter plot illustrates the relationship between the data. Meanwhile, Spearman’s coefficient’s normality plot acts in the same way as Pearson’s coefficient’s normality plot. Still, Spearman’s coefficient is applied to quantitative data, and the data does not meet the normality requirement. Kendall’s coefficient is applied to quantitative data consistency judgment, and Kendall’s coefficient is usually used to study the consistency level of scoring data (non-relational research). Pearson and Spearman correlation analyses are mainly used to calculate the correlation coefficients.

Pearson’s correlation coefficient formula:


$$\rho_{X,Y}=\frac{cov(X,Y)}{\sigma_{X}\sigma_{Y}}=\frac{E[(X-\mu_{X})(Y-\mu_{Y})]}{\sigma_{X}\sigma_{Y}}$$ (12)

The Pearson correlation coefficient is obtained by estimating the covariance and standard deviation of the sample.


$$r=\frac{\sum_{i=1}^{n}\left(X_{i}-\overset{―}{X})(Y_{i}-\overset{―}{Y}\right)}{\sqrt{\sum_{i=1}^{n}{\left(X_{i}-\overset{―}{X}\right)}^{2}}\sqrt{\sum_{i=1}^{n}{\left(Y_{i}-\overset{―}{Y}\right)}^{2}}}$$ (13)

Spearman’s correlation coefficient $r_{s}$ is calculated using the formula:


$$r_{s}=\frac{\sum \left(R_{X}-\overset{―}{R_{X}})(R_{Y}-\overset{―}{R_{Y}}\right)}{\sqrt{\sum {{(R}_{X}-\overset{―}{R_{X}})}^{2}}\sqrt{\sum {{(R}_{Y}-\overset{―}{R_{Y}})}^{2}}}=\frac{\sum R_{X}R_{Y}-\frac{(\sum R_{X})(\sum R_{Y})}{n}}{\sqrt{(\sum {(R^{2}}_{X}-\frac{({\sum R_{X})}^{2}}{n})}\sqrt{(\sum {(R^{2}}_{Y}-\frac{({\sum R_{Y})}^{2}}{n})}}$$ (14)

Compared with the formula of the Pearson correlation coefficient, the Spearman correlation coefficient only replaces X and Y in the formula of the Pearson correlation coefficient with RX and RY. It determines whether there is a correlation by calculating the value of the Pearson or Spearman correlation coefficients, and when the r is close to 1. It means that the data samples are highly positively correlated. When the r is close to −1, then it means that the data samples are highly negatively correlated. When r is close to 1, the data samples exhibit a high positive correlation; when r is close to −1, it indicates a high negative correlation; and when r is close to 0, the samples do not exhibit a correlation.

To determine that the sample is representative and valid, the decision was made to test the conclusion and assess significance, i.e., to determine importance by observing the P-value. If the overall sample X obeys normal and t distributions, its distribution curve is symmetric about the vertical axis, so its p-value can be expressed as  P=P{|X|>C. If p ≥ 0.05, the correlation result is not statistically significant, i.e., such a correlation may exist only in this sample and may not necessarily hold in the other samples; if p < 0.05, then it is called a significant correlation; if p < 0.01, then it is called a highly significant correlation.

## 4. Modeling study

### 4.1. Description of past global temperature levels and projections of future global temperature levels

The entry point for determining whether the global temperature rise in March 2022 is more significant than the change in any previous decade is to examine past trends in global temperature levels. This is done by modeling the global average temperature growth rate for any year before 2022 compared to the year before that:


$$r_{i}=\frac{{(x}_{i}-x_{i-1})}{x_{i-1}}$$ (15)

where ${\text{x}}_{\text{i}}$ is the temperature in year i, and ${\text{x}}_{\text{i}-\text{1}}$ is the temperature of the previous year of year i, and ${\text{r}}_{\text{i}}$ is the growth rate of i.

**Fig 3:**
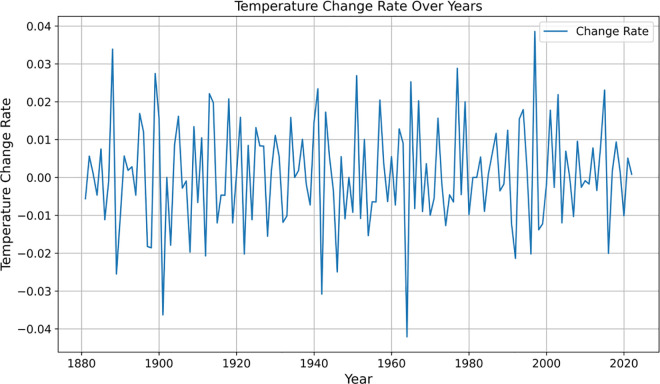
Visualisation of global average temperature growth rate data for different periods.

The global average temperature data from 1880 to 2022 were substituted into the model (2) to calculate the global average temperature growth rate data for different periods. Among them, the top five data are the 1963 global average temperature growth rate of 0.043948, the 1900 global average temperature growth rate of 0.037649, the 1941 global average temperature growth rate of 0.031776, the 1988 global average temperature growth rate of 0.026157, and the 1945 global average temperature growth rate of 0.025584. The fluctuations in the global average temperature growth rate data are visualized in Fig 3.

Fig 3 above illustrates the trend in the annual growth rate of the global average temperature from 1880 to 2022. Combined with the data on the top five global average temperature growth rates and the calculation results in Fig 3, it can be seen that the annual temperature growth rate exhibited large fluctuations in some years, such as 1963, 1900, and 1941. The temperature rise in these high-growth years was relatively rapid, suggesting that there may have been strong natural or human intervention factors at play during those years. In contrast, the negative growth in 2022 (−0.084%) indicates that the temperature in that year decreased from the previous year, reflecting the moderating effect of the short-term decline in global greenhouse gas emissions following the pandemic on the temperature rise. This figure intuitively supports the judgment of this paper that “although the growth rate of global temperature is increasing in general, there is a callback in local stages”, and provides a data basis for the subsequent analysis of the epidemic effect.

**Fig 4:**
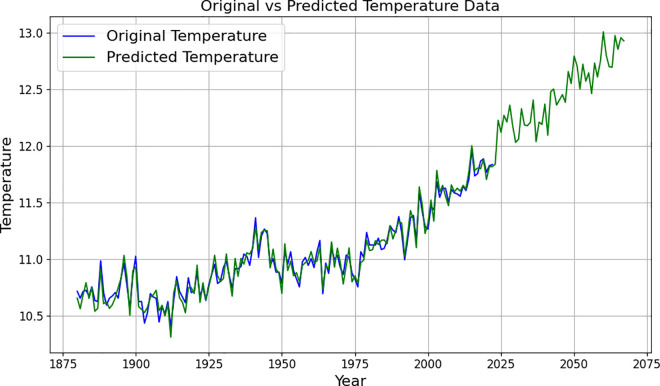
Global temperature level data for 1880-2075.

To realise the description of past global temperature levels and the prediction of future global temperature levels, the data are substituted into the LSTM model, and the obtained training results are shown in Table 4 and Fig 4 below.

**Table 4:** LSTM model – Past global temperature level data and projected future global temperature level data.

Year	Original data	Predicted data
1880	10.71444	10.71282429
1881	10.65444	10.64006652
1882	10.71444	10.71861968
1883	10.72444	10.73766426
1884	10.67444	10.65471213
…	…	…
2000	11.26444	11.31664436
2001	11.46444	11.52022701
2002	11.43444	11.33536418
2003	11.68444	11.78200933
2004	11.54444	11.5920984
...	...	...
2018	11.86444	11.79733997
2019	11.88444	11.88004742
2020	11.76444	11.70256815
2021	11.82444	11.81533655
2022	11.83444	11.81630773

Note: The data here is incomplete, and the results are for the first five groups.

Fig 4 presents the fitting and prediction results for the global average temperature from 1880 to 2075, based on the LSTM neural network model. It can be seen from the figure that the fitting effect of the model is relatively ideal in the historical data interval, and the predicted part of the curve continues to rise, indicating that LSTM successfully captures the long-term dependence and nonlinear characteristics of temperature change. The forecast results indicate that the global temperature is expected to rise to 12.79°C by 2050 and 13.97°C by 2075, exhibiting an accelerating upward trend. This predicted trend is higher than the ARIMA model results, which emphasises the advantages of LSTM in dealing with complex dynamic trends and provides a more sensitive reference for judging the global warming path.

**Fig 5:**
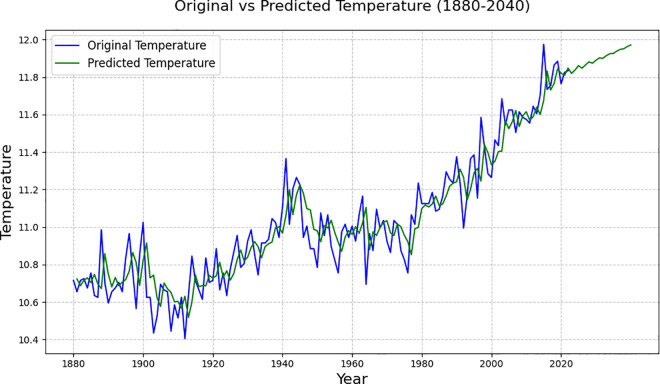
Global temperature level data, 1880-2040. Note: Serial numbers replace years in the charts; the starting year is 1880.

The ARIMA model was then applied, setting the starting year to 1880 and training the data. The results are shown in Table 5 and Fig 5. The graphs reveal the increasing temperature trend over time and predict how this trend will continue until 2040. Fig 5 illustrates the predicted global average temperature results from 1880 to 2040 using the ARIMA model. It can be observed from this figure that the ARIMA model exhibits good smoothness and consistency in fitting historical temperature data. The predicted results also indicate that the global temperature will continue to rise slowly in the future, which aligns with the conclusions of the IPCC and other climate assessment reports. Compared to the LSTM model, the ARIMA model places more emphasis on the linear trend structure; therefore, this figure is primarily used as a reference tool for assessing the robustness of the predicted trend in this study. Fig 5 further verifies the effectiveness of the ARIMA modelling method used in this study for medium- and long-term temperature trend prediction, and complements the LSTM results.

**Table 5:** Past global temperature level data and projected future global temperature level data under the ARIMA model.

Year	Original data	Predicted data
1880	10.71444	10.71282429
1881	10.65444	10.64006652
1882	10.71444	10.71861968
1883	10.72444	10.73766426
1884	10.67444	10.65471213
…	…	…
2000	11.26444	11.33188453
2001	11.46444	11.35063031
2002	11.43444	11.40205322
2003	11.68444	11.40494948
2004	11.54444	11.56752719
...	...	...
2018	11.86444	11.76599339
2019	11.88444	11.84387801
2020	11.76444	11.82030315
2021	11.82444	11.80960786
2022	11.83444	11.84825006

Note: The data here is incomplete, and the results are for the first five groups.

### 4.2. Selection of a better model

**Fig 6:**
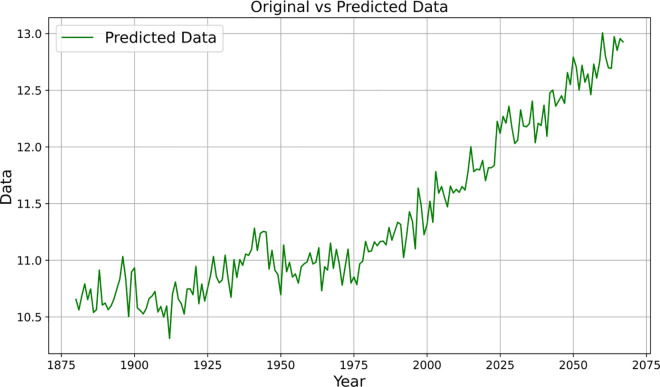
Prediction results of global average temperature change values under LSTM modelling.

**Fig 7:**
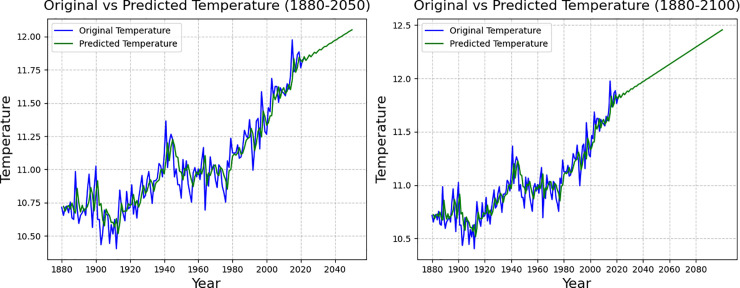
Results of 2050 projections under the ARIMA model (left), Results of 2100 projections (right).

It is observed that the prediction trends of the LSTM model and the ARIMA model are roughly the same; however, there are also gaps to explore in terms of prediction accuracy. In this paper, we use the data that predicts global temperature change in 2050 and 2100 as the starting point and substitute it into more than two different models. The results are shown in Table 6, Figs 6 and 7 below.

**Table 6:** Results of data predicted by LSTM and ARIMA models for 2050 and 2100, respectively.

Year	Predicted data in LTSM	Predicted data in ARIMA
2050	12.79	12.051
2100	14.51	12.45

Based on the model predictions, it can be seen that neither model initially predicted that the global mean temperature would reach 20°C in either 2050 or 2100. However, by extending the period and setting a target value of 20°C, the LSTM model predicts the year 2268, and the ARIMA model predicts the year 2451.

To compare the performance of LSTM and ARIMA models, the evaluation data is introduced. From the model evaluation data, the LSTM model is compared with the ARIMA model. To verify the model’s ability to capture sudden shocks, the RMSE of LSTM is 0.072, which is significantly lower than that of ARIMA’s 0.151. At the same time, the R^2^ of LSTM in the test set is 0.939, and that of ARIMA is 0.865, indicating that LSTM has better model generalisation ability than ARIMA. In general, the LSTM model performs better in terms of goodness of fit and prediction accuracy. At the same time, by introducing other classical forecasting models, such as GM (1,1), random Senna regression, ridge regression, and comparing with LSTM and ARIMA model performance, we find that other classical forecasting models are not as good as LSTM and ARIMA model in terms of goodness of fit and prediction accuracy. The results indicate that LSTM and ARIMA models exhibit better performance in predicting global temperature change, as illustrated in Table 7.

**Table 7:** LSTM and ARIMA prediction model evaluation table.

	R^2^	RMSE	MSE
LSTM	0.939	0.086	0.067
ARIMA	0.865	0.134	0.018
GM (1,1)	0.743	0.180	0.032
Random Forest regression	0.836	0.124	0.015
Ridge regression	0.739	0.181	0.033

Combined with the global status quo, i.e., the increase in global average temperature may lead to glacier melting, sea level rise, inundation of small island states, increase in extreme weather events, and even changes in the pattern of droughts and floods, which will result in the deterioration of the environment and worsening of the trend of the increase in the global average temperature, and then conduct a comparative analysis of the two models, the ARIMA model combines the results of the previous data forecasts and the fitting effect to see that the data forecasts and fitting are both better. The fitting is relatively excellent, but combined with today’s situation, the global average temperature continues to rise due to further aggravation of environmental damage, leading to a global average temperature increase of 20 °C points ahead of schedule. The LSTM model can be done according to the previous trend of the growth of the global average temperature to memorize, and in the follow-up process of the analysis, continue to strengthen this trend, and therefore ultimately believe that the LSTM model can be a more accurate prediction of the global average temperature of Therefore, it is concluded that the LSTM model can more accurately predict the change of global average temperature and its prediction results are more informative.

### 4.3. Causes affecting global temperature change

(1) Temperature change in latitude, longitude, and year

Correlation analysis was employed to investigate the primary factors influencing global temperature change. The normality of the latitude and longitude data was first tested, and the test results are presented in Table 8 below.

**Table 8:** Normality test for latitude and longitude.

Variable name	Sample size	Median	Mean value	Standard deviation	Partial degrees	Kurtosis	S-W test	K-S test
Average Temperature	19932	19.664	18.184	7.533	−0.368	−1.155	0.923 (0.000***)	0.138 (0)
Latitude	19932	28.13	22.534	21.955	−0.876	0.227	0.934 (0.000***)	0.119 (0.000)
Year	143	1951	1951	41.425	0	−1.2	0.955 (0.000***)	0.06 (0.000)
Longitude	19932	45	43.713	64.956	−0.555	−0.562	0.944 (0.000***)	0.122 (0.000)

Note: ***, **, and * represent 1%, 5%, and 10% significance levels, respectively.

Observation and analysis of the data in the above table indicate that the K-S value is too low in the normality test, which suggests that the mean temperature, longitude, and latitude are not normally distributed. This result indicates that it is suitable for correlation analysis using the Spearman correlation coefficient. The results obtained from further analysis are shown in Tables 9 and 10 below. The analysis of the results in the figure shows that the correlation between longitude and mean temperature is low. In contrast, latitude and temperature exhibit a highly negative correlation, indicating that the higher the latitude, the lower the temperature tends to be. The year shows a highly positive correlation with the temperature, indicating that the temperature increases with the year.

**Table 9:** Temperature-latitude correlation coefficient table.

	Average Temperature	Latitude	Longitude
Average Temperature	1 (0.000***)	−0.706 (0.000***)	0.108 (0.000***)
Latitude	−0.706 (0.000***)	1 (0.000***)	0.018 (0.011**)
Longitude	0.108 (0.000***)	0.018 (0.011**)	1 (0.000***)

Note: ***, **, and * represent 1%, 5%, and 10% significance levels, respectively.

**Table 10:** Temperature-year correlation coefficients.

	Tem	Year
Tem	1 (0.000***)	0.867 (0.000***)
Year	0.867 (0.000***)	1 (0.000***)

(2) Relationship between temperature change and COVID-19

This paper uses time series model projections to analyse the impact of COVID-19 on global average temperature. The first modelling assumption is made: the new crown epidemic has a slowing effect on global warming. Secondly, using the sample before 2019 to carry out time prediction, predicting the global average temperature data after 2019, if the predicted data results obtained by using the model are more significant compared to the actual results, then it indicates that the natural disaster of the New Crown Pneumonia outbreak has a specific mitigating effect on the global warming, i.e., the hypothesis is valid.

We substitute the global absolute temperature data from 2020 into the ARIMA time series model. The prediction results show that the actual value in 2020 is 11.76444, and the predicted value is 11.87497. The exact value in 2021 is 11.82444, and the predicted value is 11.89774. The actual value in 2022 is 11.83444, and the predicted value is 11.9205. A comparison and analysis of the above data reveals that the exact value is lower than the predicted value, indicating that the hypothesis that the new coronavirus epidemic has a slowing effect on global warming is valid. This paper speculates that the reason for this is that the COVID-19 pandemic led to a significant reduction in worldwide travel and energy production, indirectly resulting in a decrease in greenhouse gas emissions, which has slowed down global warming.

(3) Relationship between temperature change and greenhouse gases

To verify the accuracy of the speculation presented in the appeal, this paper selected carbon dioxide (CO_2_), sulfur hexafluoride (SF_6_), nitrous oxide (N_2_O, also known as nitrous oxide), and methane (CH_4_) among the greenhouse gases for further study and analysis.

**Fig 8:**
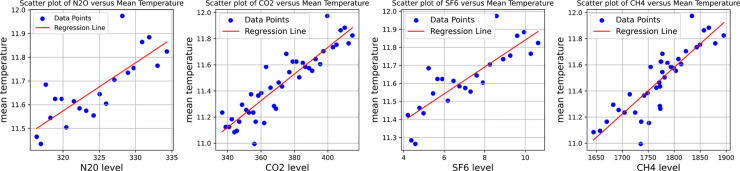
$\text{C}{\text{H}}_{\text{4}}$, ${\text{N}}_{\text{2}}\text{O}$, ${\text{SF}}_{\text{6}}$, ${\text{CO}}_{\text{2}}$ Scatterplot of temperature-dependent.

**Fig 9:**
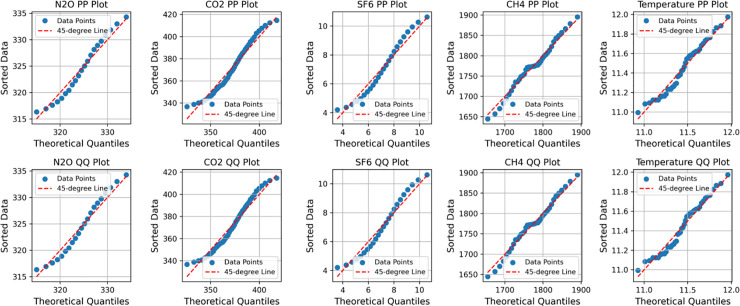
$\text{C}{\text{H}}_{\text{4}}$, ${\text{N}}_{\text{2}}\text{O}$, ${\text{SF}}_{\text{6}}$, ${\text{CO}}_{\text{2}}$, plot of normality test for temperature.

This is accomplished through the mapping of the $H_{4}$, the $N_{2}O$, ${SF}_{6}$, the ${CO}_{2}$ versus temperature correlation scatter plots (Fig 8), it was observed that greenhouse gases show a certain correlation with temperature. Based on this, the test of stationarity is then performed, which is represented by the P-P plot and Q-Q plot, respectively $CH_{4}$, the $N_{2}O$, ${SF}_{6}$, and ${CO}_{2}$, and the results are shown in Fig 9 below.

**Fig 10:**
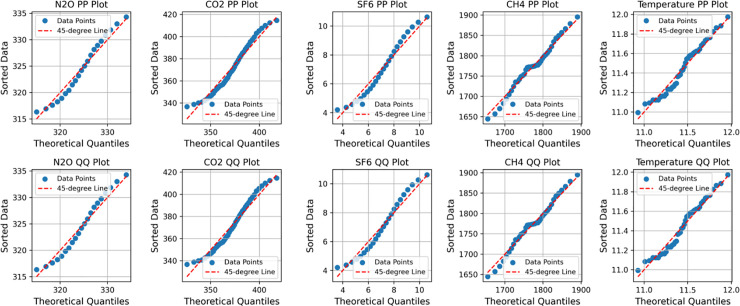
Heat map of the correlation between greenhouse gases and global temperature.

Through observation $\;CH_{4}$, $N_{2}O$, ${SF}_{6}$, ${CO}_{2}$, the P-P plot and Q-Q plot of the TEM, it is found that the scatter points and straight lines in the plot all have a high degree of overlap, which is in line with the law of normal distribution.It is suitable to use the Pearson correlation coefficient for correlation analysis. Combined with Table 11 and Fig 10, it is found that $H_{4}$, $N_{2}O$, ${SF}_{6}$, and ${CO}_{2}$ and temperature have a high positive correlation. It is evident that greenhouse gases have a significant impact on the temperature, and simultaneously, it is confirmed that they are one of the primary factors contributing to global temperature change.

**Table 11:** Table of correlation coefficients.

	${\mathrm{\backslash emph}CO}_{\mathrm{\backslash emph}2}$	${\mathrm{\backslash emph}SF}_{\text{6}}$	$\mathrm{\backslash emph}{CH}_{\text{4}}$	${\mathrm{\backslash emph}N}_{\text{2}}\mathrm{\backslash emph}O$	TEM
${CO}_{\text{2}}$	1 (0.000***)	0.999 (0.000***)	0.975 (0.000***)	0.999 (0.000***)	0.8 (0.000***)
${SF}_{\text{6}}$	0.999 (0.000***)	1 (0.000***)	0.98 (0.000***)	1 (0.000***)	0.8 (0.000***)
$CH_{\text{4}}$	0.975 (0.000***)	0.98 (0.000***)	1 (0.000***)	0.979 (0.000***)	0.794 (0.000***)
$N_{\text{2}}O$	0.999 (0.000***)	1 (0.000***)	0.979 (0.000***)	1 (0.000***)	0.801 (0.000***)
TEM	0.8 (0.000***)	0.8 (0.000***)	0.794 (0.000***)	0.801 (0.000***)	1 (0.000***)

Note: ***, **, and * represent 1%, 5%, and 10% significance levels, respectively.

**Fig 11:**
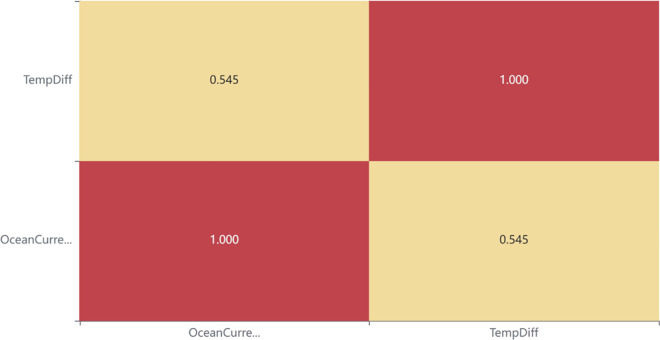
Heat map of the correlation between global temperature and ocean currents after first difference.

At the same time, to explore the influence of other factors on global temperature, the correlation between ocean currents and global temperature change is also examined to identify the years when the El Niño and La Niña phenomena occur. At the same time, a first-order differential conversion is applied to the temperature data to more accurately capture the temperature change. Through the observation and analysis of the Pearson correlation table and heat map of the first-order difference data of ocean current and global temperature, the heat map is shown in Fig 11 below. It is found that the change in global temperature is generally correlated with the state of ocean currents, indicating that the influence of ocean currents on global temperature is not more significant than that of greenhouse gases.

## 5. Conclusion and discussion

### 5.1. General discussion

This study aimed to provide an in-depth investigation of the trend of global temperature change and its complex relationship with socio-economic activities by combining LSTM and ARIMA models, as well as Pearson and Spearman correlation coefficient analyses. This study further analyses and forecasts the global average temperature data from 1880 to 2022, mainly using advanced time series analysis methods and machine learning techniques. The results show that although the global temperature increase growth rate has slowed down recently, the long-term upward trend remains significant and is highly correlated with latitude and greenhouse gas concentration. The results help us better understand the complexity of climate change and provide a scientific basis for developing effective mitigation and adaptation strategies.

### 5.2. Theoretical contributions

This study has the following three theoretical contributions:

First, it enriches the literature related to global climate change. Previous studies have confirmed the link between greenhouse gas emissions and rising global temperatures. However, few studies have comprehensively considered the impacts of socioeconomic activities, such as the potential impacts of the COVID-19 epidemic on global temperatures. In particular, the global average temperature, as an essential indicator of external societal changes, such as those affecting the natural environment and climate, has not been further discussed and investigated by scholars regarding its relevance to socio-economic activities. Therefore, this study responds to recent calls from scholars to strengthen the importance of global mean temperature research and uncovers novel associations between temperature change and socioeconomic activities.

Second, it advances research in the field of climate prediction modelling. On the one hand, the study expands the application of time series prediction models in environmental sciences by comparing the prediction effects of LSTM and ARIMA models. On the other hand, the study illustrates the correlation between the global average temperature change and various factors, including the complex relationship between latitude, longitude, and greenhouse gas concentration. Additionally, this study offers new analytical tools and methods for the field of climate science by combining time series analysis and machine learning techniques. This enhances the accuracy of climate prediction and offers insights for analysing complex systems in other fields.

Finally, new perspectives on global temperature change research are presented. In conjunction with the COVID-19 outbreak, a special socioeconomic event, this study provides a new theoretical framework for understanding global temperature change and a new research avenue for assessing the impacts of socioeconomic activities on global temperature change, as well as inspiring other researchers to consider the roles of non-conventional factors in global environmental change, and better encouraging cross-disciplinary research for a more comprehensive understanding of climate change’s complexity of climate change.

### 5.3. Practical implications

The findings of this study provide an essential guide for developing prevention and control strategies and preventive measures to address global climate change. It is known through the results of the study that:

(1) The growth rate of global temperature rise has slowed down in recent years. This indicates the effectiveness of existing global warming prevention and control measures. This finding provides confidence and direction for enterprises and governments in formulating long-term environmental policies and sustainable development strategies. Therefore, countries should continue to strengthen international cooperation and policy implementation to ensure the long-term effectiveness of these measures and identify areas for further improvement. Strengthening international cooperation can facilitate the sharing of technology and information, thereby enhancing the global capacity to respond to climate change. In addition, raising public awareness and participation is likewise crucial to maintaining effective prevention and control. More people can be made aware of the seriousness of climate change and the critical role of individuals in it through education and media campaigns [72].
(2) The trend of rising global temperatures has been significant for a long time, and the momentum of global temperatures will continue unabated. This suggests that current measures will not be able to meet future challenges in a sustainable manner. Therefore, in preventing and controlling global temperature rise, countries should not be confined to existing environmental protection policies but continue to innovate and develop to emphasise and strengthen innovative prevention and control strategies of global warming. For example, improving energy efficiency standards can reduce energy consumption and greenhouse gas emissions [73]; increasing investment in clean energy can promote the development and application of renewable energy technologies; and developing new technologies to reduce greenhouse gas emissions, such as carbon capture and storage (CCS), can help to realize deep emissions reductions in the industrial and energy sectors [74]. Implementing these measures will help build a greener and more sustainable global economy.
(3) There is a correlation between global temperature and latitude, longitude, and greenhouse gases, and the analysis of the impact of COVID-19 on the global average temperature concludes that greenhouse gases have a highly positive correlation with temperature. This conclusion suggests that changes in the ecological environment will have a direct impact on socio-economic activities. Therefore, we should adopt an interdisciplinary approach to comprehensively assess the effects of climate change on the economy and develop corresponding adaptation strategies. At the same time, it is necessary to strengthen the coordination between climate policy and economic policy to promote the realisation of a win-win situation between economic development and environmental protection. Among these, green technology and sustainable production methods can be adopted to reduce greenhouse gas emissions, promoting economic growth and social development.

In summary, the significance of this study is that it provides a new perspective to deeply understand the global temperature change trend and its interaction with socio-economic activities and builds a comprehensive analytical framework that can be used to assess the global temperature change and its relationship with socio-economic activities, which demonstrates that the global temperature change is a complex system that is influenced by multiple factors, including the impacts of natural factors and human activities. Therefore, this study provides a scientific basis for policymakers to formulate effective climate change response strategies and offers new research directions and methodologies for academics.

### 5.4. Limitations and prospects

(1) Limitation

In this study, although we used advanced time series analysis methods and machine learning techniques to gain insight into global temperature trends and their complex relationships with socio-economic activities, there are still some limitations. Firstly, the analysis of greenhouse gases, latitude and temperature is only based on statistical correlation, without in-depth verification of causality and interaction mechanism. This makes our understanding of the relationship between the three possible biases, affecting the accuracy of the model prediction and reducing the applicability of the model in different regions and climatic conditions. For example, there may be unconsidered factors perturb the GHG temperature relationship in a specific geographic area, leading to bias in model predictions. Secondly, the interaction between the global climate system and economic activities is complex and multi-dimensional nonlinear, and our model fails to fully consider the complexity of social and economic activities, such as industrialization, energy consumption patterns and policy changes. As a result, the reliability of the research results is reduced, the applicability of the model is limited, the temperature change trend may be underestimated or overestimated, and it is difficult to accurately apply to regions with different economic development stages and policy environments. Moreover, the inherent complexity of the climate system and external perturbations (such as volcanic eruptions or large-scale deforestation) affect prediction accuracy, and we do not fully account for these uncertainties. This seriously reduces the reliability of the prediction results, so that the model may have large deviations when applied to future forecasts or other similar climate conditions, and its practicability is limited. In addition, although this study uses the five-fold cross-validation method and uses indicators such as R-squared, RMSE and MSE to evaluate the model performance, there are still some limitations in the systematization and diversity of model validation. The effectiveness of the current model mainly depends on the stability performance in cross-validation and the fitting consistency between the predicted trend and historical temperature change. Therefore, there is still room for further optimization and exploration in future research, such as the introduction of external data and the use of multiple models for horizontal comparison. Finally, this study speculated that COVID-19 could effectively alleviate the global average temperature, although previous studies used similar methods to reach the same conclusion, but this study still lacks rigorous statistical indicators. In future studies, scholars can incorporate rigorous statistical methods and statistical indicators to further verify that COVID-19 can effectively alleviate the global average temperature.

(2) Future prospects

This paper has done necessary core research on model checking, but there are some optimization points for the verification of external sample sets and the in-depth study of horizontal comparison of other models. In order to further enhance the interpretability and generalization ability of the model results, the future research can further introduce external validation sets or set-aside verification, and conduct comparative analysis combined with real observation data. It can be extended to use more time series modeling methods (such as GRU, XGBoost, SVR, etc.) for horizontal comparison, so as to more comprehensively evaluate the advantages and scope of application of the model used in this study. At the same time, for possible exogenous shocks in the climate system (such as volcanic eruptions and extreme climate events), uncertainty analysis and robustness testing can be introduced in the future to enhance the model’s adaptability in complex climate environments.

As this study focuses on the long-term trend of temperature change, extreme climate events such as volcanic eruptions and El Niño are not treated separately. However, we are also aware that these events may have a short-term perturbation effect on temperature in a specific year. Although the LSTM model possesses a particular adaptive learning ability to mitigate the influence of local anomalies on the overall trend, it is still necessary to incorporate extreme climate events as exogenous variables into the analysis framework in future research. For example, the Volcanic Aerosol Index and the ENSO index (such as SST in the Niño 3.4 area) can be introduced to construct a dummy variable or covariate model, thereby improving the model’s explanatory power and scenario simulation ability in abnormal climate years. At the same time, Intervention Analysis or structural time series modelling methods can also be combined to assess the impact path of extreme events on global temperature trajectory more systematically, which provides methodological support for more accurate climate prediction.

Based on the research in this paper, LSTM and ARIMA models have good prediction performance, but in order to further improve the prediction accuracy and reliability, more complex hybrid models can be considered in the future, such as combining LSTM with CNN (convolutional neural network), using the local feature extraction ability of CNN and the time series modeling ability of LSTM. To achieve more comprehensive feature learning and prediction of global temperature data. The current research is mainly based on global average temperature data. In the future, it can be expanded to more fine-grained regional temperature data, combined with geographical, climatic, economic and other multi-source data, such as satellite remote sensing data, to explore the contribution of local climate feedback (such as Arctic amplification effect) in the latitude zone to global temperature, and deeply analyze the characteristics and driving factors of temperature change in different regions. Provide a scientific basis for the development of more targeted climate policies. At the same time, in order to overcome the above limitations, future studies can combine climate system models (such as CMIP6) and regional scale data to further analyze the contributions of natural and human factors to temperature change, and explore the heterogeneity of greenhouse gas effects under latitude gradients. Or use fruit inference methods, such as Granger causality test, vector autoregressive model, etc., to further explore the causal mechanism between these variables. Second, future research needs to adopt a more integrated approach that combines multi-source data and multidisciplinary knowledge to more fully understand the complex drivers and potential impacts of global temperature change. Finally, models need to be constantly updated and optimized to adapt to changing climate conditions and new scientific discoveries. Based on the improved model and data, such as the development of LSTM-ARIMA hybrid model, the former’s ability to capture the nonlinear trend and the latter’s analytical advantage of the stationary series are combined to realize model fusion and optimization. Furthermore, it simulated the change trend of global temperature under different policy scenarios to evaluate the effectiveness and potential impact of policies, and provided more operational decision support for policy makers.

## Supporting information

S1 File Data. (ZIP)
